# Molecular cloning and expression analysis of the STAT1 gene from olive flounder, *Paralichthys olivaceus*

**DOI:** 10.1186/1471-2172-9-31

**Published:** 2008-06-26

**Authors:** Eun-Mi Park, Jung-Ha Kang, Jung Soo Seo, GunDo Kim, Jongkyeong Chung, Tae-Jin Choi

**Affiliations:** 1 Biotechnology Research Institute, National Fisheries Research and Development Institute, 408-1, Sirang-Ri, Gijang-Eup, Gijang-Gun, Busan, 619-902, Republic of Korea; 2 Pathology Division, National Fisheries Research and Development Institute, 408-1, Sirang-Ri, Gijang-Eup, Gijang-Gun, Busan, 619-902, Republic of Korea; 3 Department of Microbiology, Pukyong National University, 599-1 Daeyeon-Dong, Nam-Gu, Busan, 608-737, Republic of Korea; 4 Department of Biological Sciences, Korea Advanced Institute of Science and Technology, Taejon, 305-701, Republic of Korea

## Abstract

**Background:**

Signal transducer and activator of transcription 1 (STAT1) is a critical component of interferon (IFN)-alpha/beta and IFN-gamma signaling. Although seven isoforms of STAT proteins have been reported from mammals, limited information is available for the STAT genes in fish. We isolated complementary DNA with high similarity to mammalian STAT1 from the olive flounder, *Paralichthys olivaceus*.

**Results:**

A DNA fragment containing the conserved SH2 domain was amplified by RT-PCR using degenerate primers designed based on the highly conserved sequences in the SH2 domains of the zebrafish and mammalian STAT1. The complete cDNA sequence was obtained by 5' and 3' RACE. The flounder STAT1 transcript consisted of 2,909 bp that encoded a polypeptide of 749 amino acids. The overall similarity between flounder STAT1 and other STATs was very high, with the highest amino acid sequence identity to snakehead (89%). Phylogenetic analyses reveal that flounder STAT1 is in the same monophyletic group with snakehead STAT1. Quantitative real time RT-PCR and in situ hybridization revealed that STAT1 was expressed in almost all examined organs and tissues, with high expression in gill, spleen, kidney, and heart. The accumulation of STAT1 mRNA in different developmental stages, as determined by real time RT-PCR, increased with development.

**Conclusion:**

Recent cloning of various cytokine genes and the STAT1 gene of olive flounder here suggest that fish also use the highly specialized JAK-STAT pathway for cytokine signaling. Identification of other STAT genes will elucidate in detail the signal transduction system in this fish.

## Background

Cellular responses to internal and external signals are mediated by the expression of specific genes or sets of genes, which are regulated by specific transcriptional factors [1]. Therefore, the entry of transcription factors into the nucleus is critical to their role in gene expression. Signals from cytokines and growth factors are transduced into the nucleus by the Janus kinase (JAK)-signal transducers and activators of transcription (STAT) signaling pathway [2-6]. JAK-STAT signaling is also involved in the regulation of cell proliferation, differentiation, survival, motility, and apoptosis in different organs [7].

The binding of signal molecules to their receptors initiates activation of JAKs, which increases their tyrosine kinase activity [4,8]. The activated JAKs phosphorylate tyrosine residues on the receptor, which turns into a binding site for proteins that contain phosphotyrosine-binding Src homology 2 (SH2) domains such as STATs [9]. The STATs bound to the phosphorylated receptor are then tyrosine-phosphorylated by JAKs. These phosphorylated STATs act as docking sites for other STATs, which results in dimerization. Activated STAT dimers accumulate in the nucleus, bind to consensus DNA-recognition motifs in the promoter regions of cytokine-inducible genes, and activate transcription of these genes [4,10]. STATs are also activated by non-receptor tyrosine kinases such as v-Src and receptor tyrosine kinases such as growth factor receptors [11,12].

There are seven distinct STATs in mammals (STAT1, 2, 3, 4, 5a, 5b, and 6) that participate in JAK-STAT signal transduction with different JAKs (JAK1, 2, 3, and Tyk2). STAT1, 2, 4, and 6 are expressed mainly in specific cell types and participate predominantly in host defense mechanisms; STAT1 is critical for interferon (IFN) function and innate immunity [13-15]. For example, STAT1 is selectively stimulated by IFN-gamma which has antiviral, immunoregulatory, and anti-tumor properties [14-16]. In mice lacking STAT1, all physiological functions associated with IFNs are absent, leading to a remarkable sensitivity to viral infections and other pathological agents [14,15]. This important physiological activity of STAT1 is expected to be conserved in fishes. Indeed, ectopic expression of the zebrafish STAT1 rescues IFN-induced signaling in a STAT1-deficient human cell line, indicating that the IFN/STAT1-dependent signaling pathway in mammals might be functionally and structurally conserved in fishes [17]. In support of this, hirame rhabdovirus (HRV)-infected olive flounder have leukocytes with upregulated levels of mRNA for the components of the IFN/STAT1-dependent signaling pathway, such as interferon-inducible 56 K protein (IFI56), CEF-10, and STAT3 [18].

Recently, flounder interleukin-8, IFN-alpha, IFN-beta, and type-1 cytokine receptors were cloned and characterized [19,20], which suggest the presence of STAT genes in this fish. In addition, a number of cytokines and growth factors have been cloned, and their activities have been successfully detected in rainbow trout and carp [21]. However, limited information is available on STAT1 in edible fishes, despite recent efforts to understand IFN-mediated anti-viral activity in fish. In this study, we present for the first time the cloning and expression analysis of a olive flounder STAT1.

## Results and Discussion

### Amplification of the SH2 Domain

The STAT1 molecule can be divided into seven functional domains: an N-domain responsible for dimer-dimer interactions, a coiled-coil domain responsible for protein-protein interactions, a DNA-binding domain, a linker domain implicated in transcription, an SH2 domain responsible for receptor binding and dimerization, a tyrosine phosphorylation site, and a transcriptional activation domain [22]. Among these seven domains, the SH2 domain shows the highest amino acid sequence similarity between various species and between STAT families. For example, the STAT1 SH2 domains of human and zebrafish show 82% identity.

**Figure 1:**
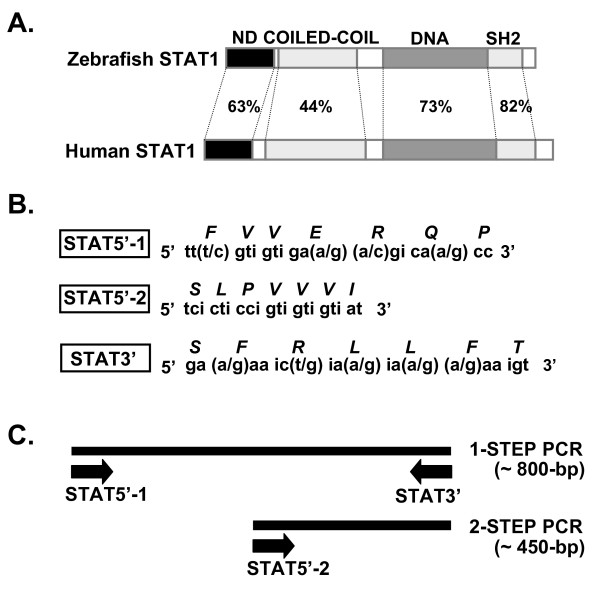
**Cloning of flounder STAT1 cDNA.** (A) Comparison of the domain structure of human STAT1 to zebrafish STAT1. ND, N-domain responsible for dimer-dimer interactions; COILED-COIL, coiled-coil domain responsible for protein-protein interactions; DNA, DNA-binding domain; SH2, src homology 2 domain responsible for receptor binding and dimerization. (B) The degenerative PCR primers used in the experiments. (C) PCR strategies used to clone flounder STAT1. The STAT5'-1 and STAT3' primers were used for first-round PCR, and the STAT5'-2 and STAT3' primers were used for nested PCR.

This structural conservation of the STAT SH2 domain in different species prompted us to clone STAT1 from *P. olivaceus*. First we constructed three degenerative PCR primers that represent three highly conserved peptide sequences within the SH2 domains of zebrafish and human STAT1, and conducted degenerative PCR reactions. The strategy for degenerative PCR used in the experiments is depicted in Fig. 1C. PCR product of 800 bp and 450 bp were obtained from first round and second round PCR reaction, respectively. As a control, parallel PCR experiments were conducted with zebrafish cDNA, and we obtained a similar result, a ~450-bp PCR product, with the same primers used for flounder (data not shown). These results strongly suggest that the 800-bp PCR product encodes a partial sequence of flounder STAT1.

To confirm that the nested PCR product is flounder STAT1, we cloned and sequenced the PCR product. The cloned cDNA was 461 bp and contained sequences that perfectly matched with the primers.

### Complete Sequence of the Flounder STAT1

The complete cDNA of the flounder STAT1 gene was compiled by overlapping the sequences of the cloned cDNA and the 5'-RACE and 3'-RACE PCR products. The flounder STAT1 transcript consisted of 2,909 bp, which translated into a 749-amino acid (aa) open reading frame (ORF) that included an 103-bp 5'-untranslated region (5'-UTR) and a 556-bp 3'-UTR. The assembled full-length cDNA sequence was entered in GenBank under accession number EF491182. The flounder STAT1 protein is the same size as that of zebrafish (749 aa), similar to that of rainbow trout (754 aa), and larger than that of crucian carp (718 aa), which is missing 39 aa at the C-terminal end. The flounder STAT1 protein contains the conserved domains of STAT proteins; the N-terminal domain (1–136), coiled-coiled domain (137–313), DNA binding domain (314–483), a linker (484–573), SH2 domain (574–679), and the transcriptional activation domain (680–749). The deduced flounder sequence contains a conserved tyrosine phosphorylation site (Y_697_) in the C-terminal activation domain. In addition, it contains the -E_422_E- and -V_451_VV- residues that are involved in DNA binding activity [23], -P_719_MSP for serine phosphorylation [24], and the arginine residue (R_599_) that is required for SH2 phosphotyrosine binding [25].

**Figure 2:**
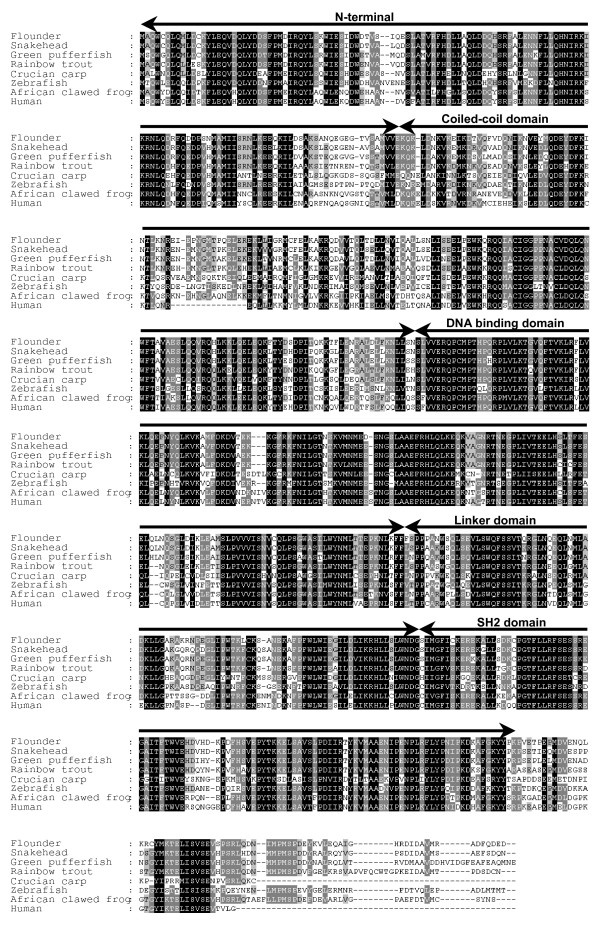
**Amino acid sequence alignments of STAT1 proteins.** Conserved amino acid residues are highlighted.

**Figure 3:**
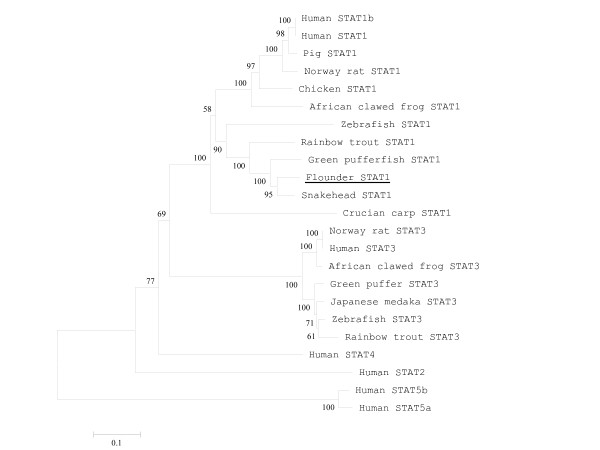
**Phylogenetic tree of the STAT family transcription factors based on amino acid sequences.** A phylogenetic tree of the aligned sequences was constructed using the neighbor-joining algorithm in MEGA (version 3.0). The confidence for each node was determined by bootstrap analysis (1000 repetitions).

The amino acid sequence of the putative flounder STAT1 was compared to known STATs from different species (Fig. 2). The overall similarity between the flounder STAT1 and other STATs was very high; snakehead STAT1 showed the highest identity (89%) and similarity (93%) at the amino acid level. Crucian carp STAT1 showed the lowest 61% identity and 77% similarity (Table 1). Figure 3 shows the phylogenetic tree based on amino acid sequence similarity. Flounder STAT1 belonged to the same monophyletic group as snakehead STAT1 (Fig. 3). Zebrafish STAT1 and human STAT1 were also included in the same monophyletic group. These results strongly indicate that the cloned DNA in this study indeed encodes the ortholog of flounder STAT1. We also note that the flounder STAT1 SH2 domain has been well conserved, and that it should play similar roles regulating STAT1 activity. As mentioned previously, zebrafish STAT1 can compensate for human STAT1 in inducing IFN-mediated signaling pathways in STAT1-deficient human cell lines [17]. Because zebrafish and flounder STAT1 have very high similarity (Table 1), we expect that flounder STAT1 also plays important roles in IFN-mediated immune activity in flounder.

**Table 1:** Amino acid sequence similarities (top-right) and identities (bottom-left) of STAT1 proteins^a^

	Human	African Clawed frog	Zebrafish	Crucian carp	Rainbow trout	Green pufferfish	Snakehead	Flounder
Human		88	77	76	82	79	80	80
African clawed frog	79		76	75	81	79	80	79
Zebrafish	62	61		72	79	78	78	78
Crucian carp	60	60	56		78	76	77	77
Rainbow trout	69	68	66	62		87	88	89
Green pufferfish	68	65	63	60	78		93	91
Snakehead	68	66	65	61	79	87		93
Flounder	68	65	64	61	79	85	89	

^a^Values are given as percentages.

### Northern Blot Analysis of Flounder STAT1 Genes

**Figure 4:**
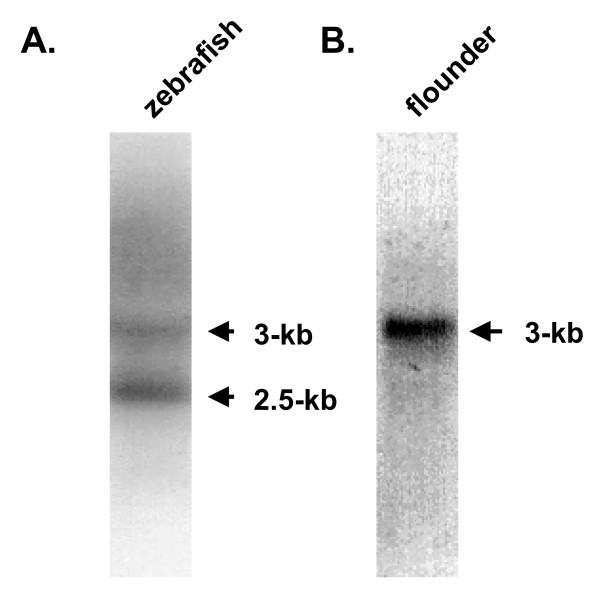
**Northern blot analysis of flounder STAT1.** (A) Zebrafish mRNA was used as a positive control that showed STAT1 (upper band) and STAT3 (lower band) mRNA. (B) Flounder mRNA detected with a flounder STAT1 SH2 domain-specific probe.

We examined the mRNA expression of flounder STAT1 by Northern blot analyses. As a control, zebrafish total RNA was analyzed using zebrafish STAT3 cDNA as a probe. As shown in Fig. 4A, we detected bands of approximately 2.5 and 3 kb. According to a previous report, the former corresponds to STAT3 and the latter to STAT1 in zebrafish [17]. Cross-reactivity of STAT3 with STAT1 mRNA is possible because the probe was derived from a 465-bp PCR fragment from the conserved SH2 domain, which has 64.7% nucleotide sequence identity between STAT1 and STAT3 [17]. Similarly, we examined STAT1 expression in flounder and observed a single ~3-kb message, which is almost the same size as the zebrafish STAT1 [17]. Considering the high sequence similarity in the SH2 domain of zebrafish STAT1 and STAT3, we expected see an mRNA band for STAT3 but did not detect another band (Fig. 4B). Although seven STAT genes have been identified in mammals [3], only STAT1, STAT3, and STAT5 have been identified in zebrafish [17,26], STAT1 in rainbow trout and crucian carp [27], and STAT5 in pufferfish [28]. In zebrafish, only cDNA clones containing STAT3 were detected using a probe derived from STAT1. The presence of different forms of STAT genes in other fish but failure in the detection of STAT3 with the conserved SH2 domain probe in flounder suggests the possibility of finding more STAT genes in flounder with low nucleotide sequence similarity.

### Expression of Flounder STAT1 in Different Tissues and Developmental Stages

**Figure 5:**
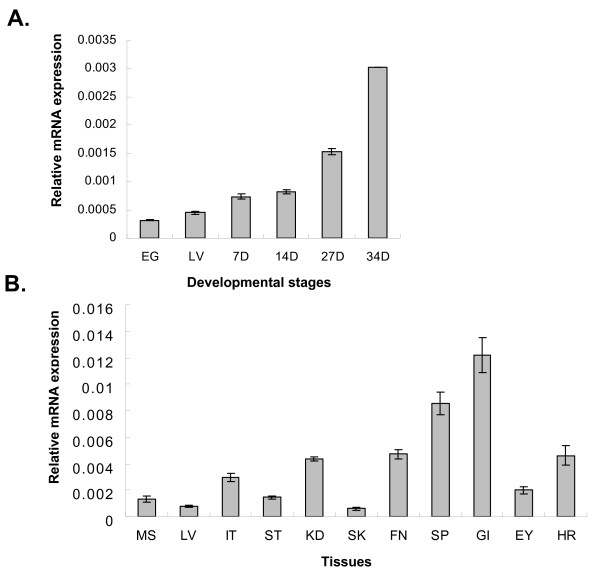
**Quantitative real-time PCR analysis of the STAT1 gene expression (A) Expression analysis of STAT1 mRNA at different developmental stages.** EG, egg; LV, larva; 7D, 7 day post-hatch; 14D, 14 day post-hatch; 27D, 27 day post-hatch; 34D, 34 day post-hatch. (B) Expression of STAT1 mRNA in various tissues of the flounder. MS, muscle; LV, liver; IT, intestine; ST, stomach; KD, head kidney; SK, skin; FN, fin; SP, spleen; GI, gill; EY, eye; HR, heart. Data are averages from three replications with standard deviations.

Some STATs, including STAT3, are expressed in early stages of development, and null mutants for STAT3 in mice are embryonic lethal [29]. In contrast, STAT1 in vertebrates is dispensable for normal development [14]. Early expression of mouse STAT1 has been detected only in endothelial cells of decidual vasculature and decidual cells, which indicates maternal expression of this gene [30]. Similarly, zebrafish STAT1 gene expression was not detected by in situ hybridization before 6 days post-fertilization (dpf) [17]. As shown in Fig. 5A, flounder STAT1 expression could be detected in all developmental stages, including the fertilized egg. Also, expression of STAT1 increased in accordance with development. Increased mouse STAT1 expression has been observed from postnatal day 0 to adulthood in the cerebellum and cerebral cortex. In our experiment, total RNA was extracted from fertilized eggs and later developmental stages. Therefore, the site of STAT1 expression could not be determined and needs to be further characterized.

**Figure 6:**
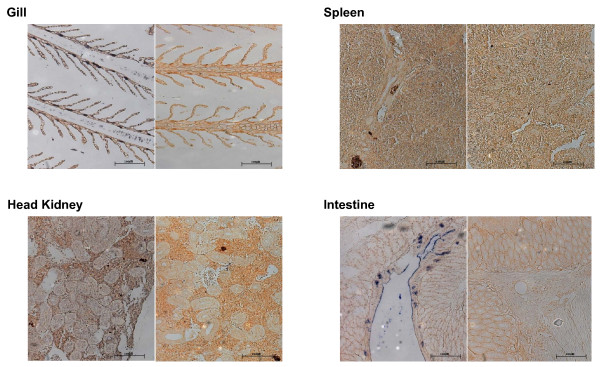
**In situ hybridization of flounder tissues to detect STAT1 mRNA.** Flounder tissues fixed in neutral buffered formalin were probed with a SH2 domain-specific cRNA probe. The left panels in each tissue hybridized with the probe show blue color, which indicates the presence of the mRNA. Right panels in each tissue showing no blue region are the same tissue probed with a sense probe as a negative control.

There are many reports of STAT1 expression in different tissues or cells of vertebrates, but studies of expression of fish STAT proteins in different tissues are limited. Sung et al. [28] observed the same expression of STAT5 in different tissues. We analyzed the expression of flounder STAT1 mRNA using quantitative real time RT-PCR and in situ hybridization. As shown in Fig. 5B, flounder STAT1 was expressed in all tested tissues. However, there was a difference in expression level; expression was low in the liver, skin, and muscle, whereas it was high in the gill, spleen and kidney. This was further confirmed by in situ hybridization, which showed high expression of STAT1 mRNA in the gill, spleen and kidney (Fig. 6). The tissues probed with SH2 domain probe showed blue appearance and there was no "hot spot" of STAT1 expression in these tissues, and STAT1 expression was detected in most cells in these tissues.

STAT1 is involved in signaling pathways initiated by both IFN-α/β and IFN-γ, which are important to innate antiviral responses and adaptive cell-mediated immune responses, respectively. In both, STAT1 is in an inactivated latent form in the cytoplasm and is activated by binding of IFNs to their receptors. Therefore, basal expression of STAT1 is necessary for a prompt response to a signal. For example, higher basal expression of STAT1 and STAT2 in cardiac myocytes results in higher antiviral protection [31]. The gill is the front line of defense when fish encounter foreign substances, including pathogenic microorganisms. Therefore, high expression of STAT1 is necessary for prompt and efficient innate immune responses. Similarly, spleen and kidney play important roles in the adaptive immune response of fish [32], and high expression of STAT1 can be expected. Our results indicate that flounder STAT1 is expressed throughout development and that tissue with high STAT1 expression are involved in defense against pathogens.

## Conclusion

Flounder is one of the main edible fishes in Asia and is also one of the most lucrative fishes in marine culture. However, many problems hamper the mass production of flounder through high-density culture. In particular, frequent mass mortality induced by viral and bacterial infections causes serious damage to the flounder industry. Therefore, biological research on the immune system of flounder is highly important, not only for academic purposes but also for commercial and industrial purposes. We cloned flounder STAT1 and found that the SH2 domain is conserved. Our results suggest that flounder may use the highly specialized JAK-STAT pathway for cytokine signaling. Further identification of IFN and isoforms of STAT proteins will provide more detail about the signaling and immune responses of this economically important fish.

## Methods

### Cloning of STAT1 cDNA in flounder

A cDNA fragment encoding the partial sequence of STAT1 was obtained by PCR from the brain tissues of olive flounder, *Paralichthys olivaceus*. A forward degenerate oligonucleotide (STAT5'-1) 5'-TT(T/C)GTIGTIGA(A/G)(A/C)GICA(A/G)CC-3' and a reverse degenerate primer (STAT3') 5'-GA(A/G)AAIC(T/G)IA(A/G)IA(A/G)(A/G)AA IGT-3' were used for first-round PCR. These primers represent two short amino acid sequences (FVVERQP and SFRLLFT) that are highly conserved in the SH2 domains of zebrafish and mammalian STAT1 (Fig. 1). Total RNA was extracted from 100 mg of flounder brain tissue. Using this RNA as a template, cDNA was synthesized using Moloney murine leukemia virus (MMLV)-reverse transcriptase and subsequently used as the template for the first-round PCR. The PCR product was subsequently amplified by nested degenerative PCR. For this second-round PCR, 5'-TCICTICCIGTIGTIGTIAT-3' (STAT5'-2) was used as a 5' degenerate primer in combination with the STAT3' primer. The successfully amplified cDNA fragment was cloned into a pGEM-T Easy vector (Promega) and sequenced.

The full-length of flounder STAT1 was obtained using rapid amplification of cDNA ends (RACE) with a SMART RACE cDNA Amplification Kit (Clontech) following the manufacturer's instructions. Total RNA (1 μg) obtained from liver was used for cDNA synthesis. The gene-specific STAT1 primers STAT1-5RACE (5'-GCAGCTGACAGACGTTAGAGATCAC-3') and STAT1-3RACE (5'-CTGAGTGACAAGTGTCCCGGCAC-3') were used in the RACE of the 5' and 3' ends, respectively. The 5' and 3' cDNA fragments obtained from RACE were cloned and sequenced as described above.

### Northern Blot Analysis

Total RNA was isolated from flounder brain and zebrafish using TRIzol reagent (Invitrogen) according to the manufacturer's instructions. The RNA samples were resolved in 1% agarose/formaldehyde gels, blotted onto Hybond-N membranes (Amersham), and hybridized in ExpressHybTM hybridization solution (Clontech). The membranes were probed with ^32^P-labeled zebrafish STAT3 cDNA or the flounder STAT1 cDNA fragment. Hybridized probes were washed in 0.1% SDS/0.1 SSC at 65°C and visualized by autoradiography.

### Structural Analysis of Flounder STAT1

The flounder STAT1 SH2 domain was identified using the TBLASTN algorithm on the BLAST server at the NCBI databank. Multiple sequence alignments were done with the CLUSTAL W algorithm in the BCM Search Launcher, and further adjusted by GeneDoc. A phylogenetic tree of the aligned sequences was constructed using the neighbor-joining algorithm within MEGA (version 3.0). The accession numbers of the sequences used in the alignments and phylogenetic tree were: olive flounder STAT1 (EF491182), snakehead STAT1 (EF079868), green puffer STAT1 and STAT3 (AF307105, AF307106), rainbow trout STAT1 and STAT3 (U60331, U60333), zebrafish STAT1 and STAT3 (NM_131480, BC068320), Japanese medaka STAT3 (AY639947), African clawed frog STAT1 and STAT3 (AY101602, AB017701), chicken STAT1 (NM_001012914), pig STAT1 (NM_213769), Norway rat STAT1 and STAT3 (AF205604, NM_012747), crucian carp (AY242386), and various human STATs (CH471058, NM_139266, NM_005419, NM_139276, NM_003151, NM_003152, NM_012448).

### Expression Studies using quantitative real-time RT-PCR

Total RNA was extracted from healthy adult *P. olivaceus *(0.5 kg in body weight), muscle, liver, intestine, stomach, kidney, skin, fin, spleen, gill, eye, and heart, or from flounder at different developmental stages using Trizol (Invitrogen). First-strand cDNA synthesis was performed using the Advantage RT-for-PCR Kit (BD Biosciences). The level of STAT1 expression was detected by RT-PCR using specific primers (STAT1-RT-F: 5'-GATCTCTAACGTCTGTCAGCTG-3' and STAT1-RT-R: 5'-GAGGTCCAGGAT TCCTTCGATC-3'). As a positive control, GAPDH was amplified using the appropriate sense (GAPDH-RT-F: 5'-TCCCATGTTCGTCATGGGCGTGA-3') and antisense (GAPDH-RT-R: 5'-ATTGAGCTCAGGGATGACCTTG-3') primers.

Reaction conditions were 94°C/4 min; 35 cycles of 94°C/30 s, 55°C/30 s, 72°C/30 s; and 72°C/10 min. SYBR^® ^Green (Molecular Probe Inc., Invitrogen) was used to detect specific PCR products. Amplification and detection of SYBR^® ^Green were performed with a MyiQ cycler (Bio-Rad). The flounder GAPDH gene was used as a housekeeping reference gene to normalize expression levels between the samples. All the data of triplicate experiments were expressed as relative to GAPDH, which was used to normalize for any difference in reverse transcriptase efficiency. Fold change in the relative gene expression to control was determined by the standard 2^-ΔΔCt ^method of Giulietti et al. [33].

### Localization of STAT1 mRNA by In Situ Hybridization (ISH)

The flounder STAT1 amplified with specific primers, STAT1-RT-F and STAT1-RT-R, was used to synthesize digoxigenin (DIG)-labeled cRNA probes. The DIG-labeled cRNA probes were synthesized using a DIG RNA labeling kit (Roche). The kidney, spleen, intestine, and gill of a healthy olive flounder were fixed in neutral buffered formalin, dehydrated, impregnated, and embedded in paraffin. After deparaffination and rehydration, 4- to 5-μm thick sections were washed for 5 min in 100% ethanol at room temperature and pretreated with Proteinase K (10 μg/ml) for 30 min at 37°C. Prehybridization and hybridization were performed in DIG Easy Hyb (Roche) solution. A hybridization mix was prepared by adding one volume of hybridization buffer to one volume of anti-sense and sense probe, giving a final concentration of at least 50 ng/μl of probe. The slides were washed with DIG wash and block buffer (Roche), and the signal was detected using the DIG Luminescent Detection Kit (Roche) according to the manufacturer's instructions. The sections were counterstained with Bismarck Brown Y before observation.

## Authors' contributions

E–MP, JSS and J–HK carried out 5' and 3' RACE, in situ hybridization, sequence alignment, real time PCR and drafted the manuscript. GK participated in 3' RACE and sequence analysis. JC carried out the cloning of the SH2 domain and southern blot analysis. T–JC participated in its design and coordination and helped to draft the manuscript. All authors read and approved the final manuscript.
